# HEPATECTOMIES: INDICATIONS AND RESULTS FROM A REFERENCE HOSPITAL IN THE BRAZILIAN AMAZON

**DOI:** 10.1590/0102-6720202400051e1845

**Published:** 2024-12-16

**Authors:** Fernanda Oliveira Barreto GARCIA, Rafael José Romero GARCIA, Mariana Pereira MAURITY, Erica Samara Monteiro NASCIMENTO

**Affiliations:** 1 Fundação Santa Casa de Misericórdia do Pará – Belém (PA), Brazil.; 2 Universidade Federal do Pará – Belém (PA), Brazil.

**Keywords:** Hepatectomy, Morbidity, Mortality, Hepatectomia, Morbidade, Mortalidade

## Abstract

**BACKGROUND:**

Hepatectomy is historically associated with higher morbidity and mortality, related to intraoperative blood loss and biliary fistulas. Technological advances and improvements in surgical and anesthetic techniques have led to greater safety in performing these surgeries.

**AIMS:**

The aim of this study was to analyze morbidity and mortality in patients undergoing hepatectomy.

**METHODS:**

Retrospective cohort study of patients undergoing liver resections. The type of hepatectomy, indications, need for intraoperative blood transfusion, hospital stay, complications, and postoperative mortality were analyzed.

**RESULTS:**

A total of 48 hepatectomies were performed during the studied period, the most common being 26 (54.16%) major hepatectomies, distributed among 13 (50%) left hepatectomies, 11 (42.30%) right hepatectomies, and 2 (7.70%) others. In total, 24 (45.84%) minor hepatectomies were performed, 11 (50%) mono segmentectomies, and 5 (22.72%) left lateral hepatectomies. The main indications for resection in benign diseases were 6 (12.50%) neotropical hepatic hydatidosis, five (10.41%) intrahepatic lithiasis, and in primary malignancies, 9 (18.75%) hepatocarcinomas. There was no need for an intraoperative blood transfusion. Hospital stays after surgery ranged from 2 to 40 days (average=7 days), and 41 (85.42%) patients went to the ICU in the first 72 h after surgery. In total, 9 (18.75%) patients developed postoperative complications. Overall mortality was 2.08%.

**CONCLUSIONS:**

Hepatocellular carcinoma and neotropical hydatidosis were the main diseases with surgical indication, and major hepatectomies were the most performed procedures. Morbidity and mortality were in line with results from major global centers.

## RESUMO

**RACIONAL:**

As hepatectomias estão historicamente relacionadas com maior morbidade e mortalidade, relacionadas a perda sanguínea intra-operatória e fistulas biliares. Os avanços tecnológicos e o aprimoramento da técnica operatória e anestésica levaram a maior segurança na realização destas cirurgias.

**OBJETIVOS:**

Avaliar a morbidade e a mortalidade em pacientes submetidos à hepatectomias.

**MÉTODOS:**

Estudo de coorte retrospectivo de prontuários de pacientes submetidos a ressecções hepáticas. Foram analisados o tipo de hepatectomia, indicações, necessidade de transfusão sanguínea intra-operatória, permanência hospitalar, complicações e a mortalidade pós-operatórias.

**RESULTADOS:**

Foram realizadas um total de 48 hepatectomias no período estudado, sendo mais comuns 26 (54,16%) as hepatectomias maiores, distribuídas entre 13 (50%) hepatectomias esquerdas; 11 (42,30%) hepatectomias direitas e 2 (7,70%) outras. Foram feitas 22 (45,84%) hepatectomias menores, sendo 11 (50%) monossegmentectomias e 5 (22,72%) hepatectomias laterais esquerdas. As principais indicações para ressecção em doenças benignas foram 6 (12,50%) hidatidose hepática neotropical; 5 (10,41%) litíase intra-hepáticae nas malignas primárias, 9 (18,75%) hepatocarcinomas. Não houve necessidade de hemotransfusão no intra-operatório. A permanência hospitalar após a cirurgia variou de 2 a 40 dias (média=7 dias); 41 (85,42%) pacientes foram para UTI nas primeiras 72 horas do pós-operatório. Nove (18,75%) pacientes desenvolveram complicações pós-operatórias. A mortalidade geral foi 2,08%.

**CONCLUSÕES:**

O carcinoma hepatocelular e a hidatidose neotropical foram as principais doenças com indicação cirúrgica, e as hepatectomias maiores foram os procedimentos mais realizados. A morbi-mortalidade está em linha com os resultados de grandes centros mundiais.

## INTRODUCTION

**Central Message**

Liver resections have historically been associated with increased morbidity and mortality, particularly in the face of diseases and complications such as intraoperative blood loss and biliary fistulas. Technological advances and improvements in surgical and anesthetic techniques have led to greater safety in performing these surgeries.

**Perspectives**

Hepatectomies performed safely, primarily for neotropical hydatidosis and intrahepatic lithiasis, resulted in a low overall incidence of postoperative complications. Therefore, in the context of public health in the north of the country, it is possible to obtain good results, with multidisciplinary teams dedicated to the treatment of liver diseases.

Liver surgery is one of the last frontiers of abdominal surgery, with great technical developments over the last three decades. Knowledge of liver segmentation based on vascularization and biliary drainage, as described by Couinaud, has allowed advances in the surgical treatment of liver diseases. Advances in anesthetic techniques and postoperative management have made surgeries safer. The incorporation of imaging tests with better definition and evaluation by specialized multidisciplinary teams has allowed for better preoperative planning^2,22^.

Hepatectomy is intended to resect liver lesions, whether benign or malignant, primary or secondary. Regarding symptoms, most benign lesions are asymptomatic, and not all require resection. The most common benign lesions are hemangiomas, focal nodular hyperplasia, and adenomas. Regarding primary malignant tumors, hepatocellular carcinoma represents 85% of these neoplasms^1,21^.

The National Cancer Institute estimates that there will be 10,700 new cases of liver cancer in Brazil between 2023 and 2025, with males being the most affected. Infection by the hepatitis B and C viruses is the main cause of this neoplasia, followed by metabolic diseases such as steatosis, nonalcoholic hepatitis, and alcoholism. According to the Hospital Information System of the Unified Health System (SIH/SUS), 442 patients were hospitalized in healthcare facilities in the city of Belém due to malignant neoplasia of the liver and intrahepatic bile ducts^14,17^.

The research proposes to study the cases treated by the State Reference Center for Liver Diseases at the Santa Casa de Misericórdia do Pará Foundation. Further, it aims to analyze the morbidity, mortality, and early surgical results of hepatectomies and identify the clinical and demographic profile of the patients who underwent this treatment.

## METHODS

This study was carried out with a retrospective analysis of the electronic medical records of the Santa Casa de Misericórdia do Pará Foundation (FSCMPA), Belém, Brazil, in search of all cases of patients who underwent liver resections, from January 2019 to December 2023. It is important to note that during the pandemic period in 2020, no elective surgeries were performed at this institution. In total, 48 medical records were selected by the Medical Archive Coordination (CAME). The project was approved by the Research Ethics Committee of the Institution (number 6.560.856).

Of the selected patients, the following information was extracted from their medical records: sex, age, type of hepatectomy, indication for liver resection, complications, need for blood transfusion, length of hospital stay, mortality up to 90 days postoperatively, use of drains, and length of drain indwelling.

Hepatectomies were mostly performed by laparotomy, but laparoscopy was also used for indications where the nodule was in a favorable position, as in some monosegmentectomies. Resection of more than three segments was considered a major resection. Liver resections were classified according to the Brisbane nomenclature system^25^.

Intraoperative ultrasonography was performed in all cases, as well as the intermittent Pringle maneuver. Energy devices, such as argon plasma, monopolar, and bipolar cauteries, were used to reduce blood loss. Parenchymal transection was always performed using the kellyclasia technique. Bile leak testing^6^ was performed whenever possible. Postoperative complications were classified using the modified Clavien–Dindo scoring system^5^.

For statistical analysis, all collected data were organized and analyzed using Python 3.8 and Microsoft Excel^®^ 365 programs. The numerical variables in the study were identified as mean and standard deviation. The chi-square test was used to find potential relationships between variables, with values considered statistically significant, and hypothesis tests with p<0.05. In addition, graphs were presented for visualization and comparison between groups of individuals.

## RESULTS

In total, 48 hepatectomies were performed during the study period. The mean age of the patients was 53.10 years, ranging from 24 to 78 years, and the majority were female. Among the sample analyzed, 41 patients required admission to the intensive care unit (ICU), following institutional protocol. In most surgeries, there was no need for placement of a drain in the abdominal cavity, and there was only one death recorded, as shown in Table 1.

**Table 1:** Distribution of patients.

Parameter	Participation
Age	53.10±14.10
Sex	Male: 18 (37.50%); female: 30 (62.50%)
ICU	Yes: 41 (85.42%) Nº: 7 (14.58%)
ICU days	1.92±3.15
Drain	Yes: 7 (14.58%) No: 41 (85.42%)
Drain time	3.15±14.88
Death	1

ICU: intensive care unit.

Most indications were for benign diseases (n=33; 68.7%); neotropical hydatidosis and intrahepatic lithiasis appeared most frequently. In malignant diseases, hepatocarcinoma appears as the main indication (Table 2). Other malignant neoplasms, such as fibrolamellar hepatocarcinoma and cholangiocarcinomas, although less frequent, denote the diversity of primary liver tumors that challenge surgical practice.

**Table 2:** Distribution of diseases that served as the basis for the indication of 48 hepatectomies, in 4 years.

Indications for hepatectomy	Frequency
Benign diseases	
Neotropical hydatidosis	6
Intrahepatic lithiasis	5
Liver nodule	6
Hepatocellular adenoma	4
Hemangioma	3
Liver cyst	3
Complex liver cyst	4
Liver mass	1
Liver abscess	1
Malignant diseases	
Hepatocarcinoma	10
Fibrolamellar hepatocarcinoma	1
Cholangiocarcinoma (liver mass)	1
Cholangiocarcinoma (bile duct tumor)	2
Polypoid mass	1
Total	48

In total, 22 (45.8%) minor and 26 (54.1%) major hepatectomies were performed, as shown in Table 3. Among the minor hepatectomies, monosegmentectomies and bisegmentectomies were the most frequent.

**Table 3:** Distribution of the 48 hepatectomies performed in 4 years, according to the type of liver resection.

Type of hepatectomy	Frequency
Minor hepatectomies	
Monosegmentectomies:	
Sec III	3
Sec IV	1
Mon V	1
Sec VI	3
Sec VII	3
Bisegmentectomies	
Sec II and III	5
Seg II and IV	1
Seg II and V	1
Seg IV and V	2
Seg IV and VII	2
Major hepatectomies	
Right	11
Right trisegmentectomy	1
Left	13
Central	1
Total	48

Seg: segment.

A comparative analysis of postoperative complications was performed according to the Clavien–Dindo classification. Complications were grouped according to their severity (Table 4). The data indicate that 81.25% of the cases analyzed did not fall into the grade I category, as they did not require any therapeutic intervention permitted in this classification grade during hospitalization, such as antiemetic drugs, antipyretics, analgesics, diuretics, electrolytes, and physiotherapy. The remaining 18.75% of complications were classified as grade II, IIB, IVA, and V as demonstrated in Table 5.

**Table 4:** Distribution of postoperative complications after 48 hepatectomies.

Complication	Frequency
Surgical wound infection	1
Perihepatic collection	1
Biliary fistula	1
Pneumothorax	1
Surgical wound dehiscence	1
Pneumonia	1
Abdominal sepsis	2
Renal failure	1

**Table 5:** Complications of patients according to the Clavien-Dindo classification.

Grading grade	Nº	%
II	3	6.25
III B	4	8.34
VAT	1	2.08
V	1	2.08

The mean length of postoperative hospital stay was approximately 6.72 days, with a standard deviation of 8.06 days. While some patients could be discharged in as little as 2 days, indicating a rapid recovery, others required up to 40 days to reach adequate conditions for discharge, demonstrating cases of more prolonged recovery. These observations are detailed in Table 6.

**Table 6:** Descriptive statistics of patient discharge time.

Variable	Value
Average days	6.72
Standard deviation	8.06
Minimum	2
Maximum	40
Death	1

The overall morbidity and mortality rates were 18.75% and 2.08%, respectively. On the contrary, the morbidity and mortality rates for patients undergoing major resections were 19.23% and zero deaths, respectively.

## DISCUSSION

The first elective liver resection was performed in Berlin by Carl Von Langenbuch in 1887^16^ to remove a liver tumor. In Brazil, the first hepatectomies date back to the 1950s^10^. However, surgical improvements began to emerge only in the last 30 years, with advances in medical knowledge, development, and improvement of surgical techniques, more detailed examinations, and safer anesthetic measures^4,11^. Given the current scenario, these multidisciplinary advances allow hepatectomies to be performed with minimal morbidity and mortality rates, which is essential to expanding access to hepatectomy^11^.

Morbidity and mortality rates related to hepatectomies are not the only ones, but they are an important indicator of the efficiency of these procedures^3^. The Clavien-Dindo classification has been progressively accepted since its creation in 2004. In addition, more recent studies have validated this classification for several types of surgery^28^. Therefore, the overall postoperative complications and mortality rates in the present study were 18.75 and 2.08%, respectively. The complications and mortality rates found here are within the world average; the mortality rate was lower than that described in recent national literature, that is, it was between 1 and 5%^23^.

The performance of a meticulous surgical technique in the parenchyma section was important in preventing biliary fistula, which shows that the incidence of this complication was only 2.08% in the present study. Thus, as in other studies, the larger vessels and bile ducts were sutured to prevent postoperative bleeding and fistulas^20,27^. The absence of the need for blood transfusion in this study also contributed to and is related to a good postoperative evolution and short hospital stay of patients who were discharged, in most cases, on the 6th postoperative day. In some reported series, patients who received one and more than two units of blood transfusion had an operative mortality of 2.5 and 11.1%, respectively, compared with 1.2% for patients who did not require transfusions^15^.

Still on complications, the present study presented three cases of surgical re-approach: one due to surgical wound dehiscence and two due to abdominal sepsis, the latter requiring exploratory laparotomy. From previous studies, bleeding and abdominal wall complications are the main indications for reoperation after hepatectomy^18^. The most common complications were infectious, including perihepatic collection, surgical wound infection, dehiscence, sepsis, and cholangitis (6/48; 12.5%), related to biliary fistula, and pneumothorax and pneumonia, related to pulmonary (2/48; 4.16%), while Feier et al. reported pulmonary and infectious complications in 39.7 and 28.7%, respectively. We observed one death in the present study, which is in accordance with the literature^8,12,19,24^.

Regarding the sample of cases and mortality rate analyzed in the present study over a period of 4 years, they were higher than in another study also carried out in the city of Belém in an analysis of a period of 10 years, presenting approximately 33 cases, with a mortality rate of 48.48% (n=16), with one death reported during surgery^26^. Comparing the mortality rate in the present study (2.08%) with that cited, a mortality rate approximately 23 times higher is observed.

The mortality found in this study in a cirrhotic patient with hepatocellular carcinoma, despite the absence of intraoperative complications, led us to understand that the indication of hepatectomy in these patients should be judicious and protocols aiming at better selection should be followed^7,13^.

A Brazilian study presented a retrospective analysis of 129 resections in non-cirrhotic livers, 42.6% of which were considered major hepatectomies^8^. Most of the cases included patients with non-cirrhotic livers (84.3–100%) and malignant disease (48—96.6%). The present study presents the prevalence of major hepatectomies and with (5/26; 19.23%) indication for major hepatectomy due to some malignant disease^9^.

Considering studies in the north of the country, a survey carried out by the digestive system oncology surgery service in the city of Manaus-Amazonas presented a sample of 34 cases of liver resection in the period of 5 years, with 11 cases of complications in the postoperative period, the most notable being respiratory infections, atelectasis, and urinary tract infections^3^. In comparison with the present study, there were nine cases of complications in the postoperative period, with abdominal sepsis being the most frequent.

## CONCLUSIONS

Safe hepatectomies, with the main indications being neotropical hydatidosis and intrahepatic lithiasis, resulted in a low overall incidence of postoperative complications. Therefore, in the context of public health in the north of the country, it is possible to obtain good results, with multidisciplinary teams dedicated to the treatment of liver diseases.
